# Microfluidic Biofabrication of 3D Multicellular Spheroids by Modulation of Non-geometrical Parameters

**DOI:** 10.3389/fbioe.2020.00366

**Published:** 2020-05-05

**Authors:** Silvia Lopa, Francesco Piraino, Giuseppe Talò, Valerio Luca Mainardi, Simone Bersini, Margherita Pierro, Luigi Zagra, Marco Rasponi, Matteo Moretti

**Affiliations:** ^1^IRCCS Istituto Ortopedico Galeazzi, Cell and Tissue Engineering Laboratory, Milan, Italy; ^2^Department of Electronics, Information and Bioengineering, Politecnico di Milano, Milan, Italy; ^3^Regenerative Medicine Technologies Laboratory, Ente Ospedaliero Cantonale, Lugano, Switzerland; ^4^Laboratory for Biological Structures Mechanics, Chemistry, Material and Chemical Engineering Department “Giulio Natta,” Politecnico di Milano, Milan, Italy; ^5^IRCCS Istituto Ortopedico Galeazzi, Hip Department, Milan, Italy

**Keywords:** microfluidic, spheroid, 3D culture, pellet culture, fluid dynamic

## Abstract

Three-dimensional (3D) cell spheroids are being increasingly applied in many research fields due to their enhanced biological functions as compared to conventional two-dimensional (2D) cultures. 3D cell spheroids can replicate tissue functions, which enables their use both as *in vitro* models and as building blocks in tissue biofabrication approaches. In this study, we developed a perfusable microfluidic platform suitable for robust and reproducible 3D cell spheroid formation and tissue maturation. The geometry of the device was optimized through computational fluid dynamic (CFD) simulations to improve cell trapping. Experimental data were used in turn to generate a model able to predict the number of trapped cells as a function of cell concentration, flow rate, and seeding time. We demonstrated that tuning non-geometrical parameters it is possible to control the size and shape of 3D cell spheroids generated using articular chondrocytes (ACs) as cellular model. After seeding, cells were cultured under perfusion at different flow rates (20, 100, and 500 μl/min), which induced the formation of conical and spherical spheroids. Wall shear stress values on cell spheroids, computed by CFD simulations, increased accordingly to the flow rate while remaining under the chondroprotective threshold in all configurations. The effect of flow rate on cell number, metabolic activity, and tissue-specific matrix deposition was evaluated and correlated with fluid velocity and shear stress distribution. The obtained results demonstrated that our device represents a helpful tool to generate stable 3D cell spheroids which can find application both to develop advanced *in vitro* models for the study of physio-pathological tissue maturation mechanisms and to obtain building blocks for the biofabrication of macrotissues.

## Introduction

Three-dimensional (3D) cell spheroids have enhanced biological functions compared to conventional two-dimensional (2D) cultures (Pampaloni et al., 2007; Fang and Eglen, 2017). In particular, self-assembled 3D cell spheroids can replicate tissue functionality, representing useful models of tumorigenesis (Kwapiszewska et al., 2014; Ishiguro et al., 2017), embryonic development (Nakazawa et al., 2013; Pettinato et al., 2014; Belair et al., 2017), and chondrogenesis (Sabatino et al., 2012; Lopa et al., 2013; Sridharan et al., 2018) that bridge the gap between classical *in vitro* studies and animal studies (Zorlutuna et al., 2012). Moreover, 3D cell spheroids are being increasingly applied as building blocks for tissue engineering applications due to the possibility of achieving *in vitro* tissue maturation before their assembly into macrotissues of desired shape by biofabrication techniques, such as bioprinting (Laschke and Menger, 2017). In this scenario, the development of platforms to achieve robust and reproducible 3D cell spheroid formation and tissue maturation appears as a crucial step to engineer advanced *in vitro* models and pave the way to tissue biofabrication.

Traditional methods for 3D cell spheroid formation include the culture on non-adhesive substrates, the use of rotating vessel bioreactors, the hanging-drop method, and the centrifugation in conical tubes. However, all these approaches are characterized by a limited control over the size and geometry of 3D cell spheroids. In the last years, several microwell platforms have been developed by microfabrication technologies to overcome this limitation (Selimovic et al., 2011; Piraino et al., 2012; Lopa et al., 2015; Lee et al., 2016), finding an important application in studies where cell function is strictly connected to the size and geometry of the 3D spheroid (Moreira Teixeira et al., 2012; Babur et al., 2013; Sridharan et al., 2015; Liu et al., 2017). These features are usually modulated by modifying the geometry of the microwells (Karp et al., 2007; Napolitano et al., 2007; Moeller et al., 2008; Sakai et al., 2010; Masuda et al., 2012), which is the main tunable parameter in static culture platforms.

Compared to static microwell systems, microfluidics offers the advantage to modulate additional parameters, such as flow rate and shear stress. The effect of these parameters is strictly dependent on the chip design. For example, it has been shown that the presence of microgrooves within microchannel strongly influences the fluid dynamic environment. Moreover, the modulation of microgrooves geometry (width and height) determines microcirculation areas and microscale shear stresses, in turn affecting cell trapping (Manbachi et al., 2008; Karimi et al., 2013; Khabiry and Jalili, 2015). However, given a fixed microfluidic chip design, the fluid flow can be tuned to obtain different fluid dynamics microenvironment, a possibility that is usually neglected in view of tuning cell trapping and 3D cell spheroid formation.

Computational fluid dynamics (CFD) modeling is a powerful tool that is being applied to assist microfluidic platforms design, allowing to unravel the factors determining specific hydrodynamic patterns, and study the influence of fluid dynamics on cell behavior (Huang et al., 2010). Interesting results have been provided by studies combining CFD simulations and experimental cell trapping, demonstrating that improved results can be achieved through the CFD-driven optimization of chip geometry (Khabiry et al., 2009; Cioffi et al., 2010) and thus proving the value of this computational-experimental approach. CFD modeling can also be exploited to investigate the effect of mechanical cues on cell behavior. For instance, mechanical factors are known to play a key role in tissue development *in vivo* (Mammoto and Ingber, 2010). Based on this, culture platforms compatible with the application of mechanical stimulation can be used to gain a better understanding of tissue maturation and exploit biophysical cues to enhance this process. In this scenario, CFD modeling is essential to interpret the experimental results and identify the biophysical determinants of cell behavior.

The aim of this study was to control cell trapping and 3D cell spheroid formation by tuning non-geometrical parameters within a perfused microfluidic environment through a computational–experimental approach. Here, articular chondrocytes (ACs) were used since chondrocytes are known as 3D spheroid-forming cells and as responsive to biophysical cues. CFD modeling was exploited to optimize chip geometry, while cell concentration, flow rate, and seeding time were modulated to control and generate a predictive model of cell trapping. CFD modeling was subsequently used to study the influence of fluid flow and shear stress on 3D cell spheroid formation and tissue maturation.

## Materials and Methods

### Study Design

In this study, we evaluated the influence of multiple non-geometrical parameters, such as cell concentration, flow rate, and seeding time on cell trapping in a concave microfluidic chamber. Computational modeling was used to predict cell trapping and correlate the formation and biological response of 3D spheroids of human ACs with the fluid dynamic features associated to different flow rates.

### Design and Optimization of the Microfluidic Chip Based on CFD Modeling

A microfluidic concave chamber was conceived, resembling the conical end-tip of a standard 1.5 ml tube routinely used to generate 3D cell spheroids. Computational modeling was used to optimize its geometry. At first, a preliminary chamber design was realized with a desktop CNC milling machine (MDX40, Roland DG) through a carbide round mill (Ø 400 μm). The chamber surface was characterized by the presence of micro-burrs. Images of the chamber were acquired using an IX71 inverted microscope (Olympus) and analyzed to reproduce the actual geometry in the numerical model. CFD modeling was exploited to verify whether a simplified (smooth) geometry of the chamber could be adopted in the simulations to replace the actual design. Chamber geometries were discretized by GAMBIT (Ansys Inc.) adopting a tetrahedral meshing scheme with about 2,000,000 elements. The steady-state Navier–Stokes equations for an incompressible fluid were then solved using the finite volume code ANSYS FLUENT (Ansys Inc.). In all the simulations, the following conditions were applied: no-slip boundary at walls, zero pressure at the outlet, and flat average velocity profiles at the inlet port. The velocity fields calculated by CFD simulations in both configurations were compared at different depths. The microfluidic chip geometry was then analyzed by CFD modeling to verify whether the inclusion of an additional groove between the microchannels and the seeding chamber could be a suitable option to reduce the flow velocity in proximity of the chamber, hence favoring cell trapping by sedimentation.

### Fabrication of the Microfluidic Device

Based on the results of the CFD simulations, the microfluidic device was designed to include a channel allowing for cell loading and medium perfusion, a groove to reduce the velocity of flowing cells, and a concave chamber for cell trapping and 3D spheroid culture.

The microfabrication consisted in the manufacturing of two layers obtained by replica molding of poly(dimethylsiloxane) (PDMS) and assembled together to obtain a continuous fluidic domain (Xia and Whitesides, 1998). The top layer mold was obtained by transferring the layout of a straight channel (40 mm long, 250 μm wide) onto a 4″ silicon wafer through photolithography, achieving a SU-8 50 (MicroChem Corp.) negative photoresist relief with a height of 150 μm. PDMS prepolymer and curing agent (Sylgard 184, Dow Corning), mixed in ratio 10:1 (w/w), were then poured on the mold, degassed, and cured in oven at 80°C for 120 min. PDMS was then demolded and inlet and outlet ports were realized through a 0.5 mm biopsy puncher. The bottom layer, including the culture chamber and the upstream and downstream groove, was obtained through a series of consecutive fabrication steps. At first, the fluidic volume of the chamber and the groove were machined on a flat 8 mm thick poly(methylmethacrylate) (PMMA) substrate with CNC milling machine (round mill, Ø 400 μm) at a final depth of 2.2 mm. PDMS prepolymer and curing agent, mixed in ratio 10:1 (w/w), were then poured on the PMMA mold, degassed, and cured in oven at 80°C for 120 min. Subsequently, the PDMS layer was removed from the mold, and its surface underwent an air plasma treatment (Harrick Plasma, United States), followed by 30 min of chloro-trimethyl-silane vapor deposition (Sigma-Aldrich) in a saturated atmosphere. PDMS was then used as mold, and fresh PDMS (10:1, w/w) was poured on top of it and cast on a hot plate at 150°C for 10 min. Finally, the obtained PDMS block was bonded to the top layer.

### Cell Isolation and Expansion

Human articular cartilage was harvested aseptically from discarded tissue fragments obtained from osteoarthritic patients (Kellgren–Lawrence grade III–IV) undergoing routine arthroplasty surgical procedures, with patients’ informed consent. ACs were isolated by macroscopically non-fibrillated regions of cartilage by enzymatic digestion of minced tissue. After digestion, cells were plated at 10^4^ cells/cm^2^ in complete medium (CM) added with 5 ng/ml FGF-2 and 1 ng/ml TGF-β1 (Lagana et al., 2014). After 2 weeks, cells were detached and suspended in CM at 5, 10, and 20 million (M) cells/ml for seeding.

### Cell Trapping

#### Experimental Set-Up

Before use, microfluidic devices were cleaned by flowing 70% ethanol at 10 μl/min for 3 min and rinsing with phosphate buffer solution (PBS), and finally filled with CM. Cells were seeded using a syringe pump (PHD 22/2000, Harvard Apparatus). Different combinations of cell concentrations (5M, 10M, and 20M cells/mL), flow rates (1.25, 2.5, and 5 μl/min), and seeding times (10, 20, and 40 min) were tested as non-geometrical parameters able to modulate cell docking.

#### Generation of a Predictive Model for Cell Trapping

A statistical software (JMP, SAS Institute Inc.) was used to analyze the interactions between the analyzed non-geometrical parameters and to predict their effect on cell trapping. The number of trapped cells represented the output, whereas cell concentration, flow rate, and seeding time represented the input variables. All the experimental conditions and the relative number of trapped cells were included in the model. Experiments in an additional condition (i.e., 10M cells/ml, 2.5 μl/min, 60 min) were performed to test the model using the simulation tool provided by JMP with the following specifications: random error, five repetitions. The interaction plot and the correlation plot were drawn using the software to evaluate the interaction between cell concentration and flow rate or seeding time and to compare experimental and predicted results.

### Dynamic Generation and Culture of 3D Cell Aggregates

#### Experimental Set-Up

3D cell aggregates were generated within the microfluidic device. The following parameters were used for the seeding phase: 10M cells/ml, 2.5 μl/min, 20 min. After seeding, devices were incubated at 37°C, 5% CO_2_ and perfused with serum-free chondrogenic medium (Lopa et al., 2013) using an intermittent flow rate (1 min perfusion each hour). Three flow rates were tested: 20, 100, and 500 μl/min. Samples were cultured for 14 days.

#### Modeling of 3D Cell Spheroids Generated Under Different Flow Rates

Computational models were gleaned on the different aggregate geometries generated upon the tested flow conditions. Specifically, three models were developed: a filled chamber, a disk-shaped aggregate, and a spherical aggregate. The filled chamber model was generated creating a 3D surface above the concave chamber. The other models were developed by designing solid bodies inside the chamber: a disk (Ø_*max*_ 1.9 mm, height 1.1 mm) or a solid sphere (Ø 1 mm).

### Sample Analysis

#### DNA Assay

Immediately after seeding, the top layer of the chip was punched (Ø 2 mm) and cells trapped in the concave chamber were retrieved using a micropipette. Similarly, cell aggregates were retrieved from the microfluidic devices after 14 days of culture and digested with papain (60°C, 16 h). DNA quantification was performed by CyQUANT kit, according to the manufacturer’s instructions. Fluorescence was measured (λ_ex_ 485 nm–λ_em_ 535 nm) using a Victor X3 Plate Reader (Perkin Elmer).

#### Alamar Blue Assay

Cell metabolic activity was assessed through the Alamar Blue^TM^ cell viability assay (Life Technologies). Briefly, cell spheroids were transferred to a 96-well plate and incubated with 10% Alamar Blue^TM^ in HG-DMEM (37°C, 4 h). After incubation, the supernatant was harvested and fluorescence was measured (λ_ex_ 540 nm–λ_em_ 580 nm) using a Victor X3 Plate Reader. Fluorescence values were then normalized on the DNA content to obtain the metabolic activity per cell, defined as normalized metabolic activity.

#### Glycosaminoglycan Assay

After papain digestion, GAG content of cell aggregates was measured by dimethylmethylene blue assay (16 mg/ml, Sigma-Aldrich) with chondroitin sulfate as standard. Absorbance was read (λ 525 nm) using a Victor X3 Plate Reader.

#### Histological Evaluation

Cell aggregates were fixed in 4% neutral buffered formalin, dehydrated in a graded series of alcohols, individually embedded in paraffin, and sectioned at 4 μm. Sections were stained with hematoxylin and eosin to visualize cell and extracellular matrix distribution and with alcian blue to detect GAG deposition. Images were acquired using an Olympus IX71 microscope.

### Statistical Analysis

Data are presented as mean + standard deviation (SD), except where otherwise noted. Each experimental condition was repeated at least three times (*n* ≥ 3). Data were analyzed using GraphPad Prism. Data distribution was evaluated by means of D’Agostino and Pearson normality test. Comparison between two groups was performed by *t*-test for parametric or non-parametric data, depending on data normality. Comparison of multiple groups was performed by univariate analysis of variance (ANOVA) followed by Bonferroni’s or Dunnet’s *post hoc* tests for parametric and non-parametric data, respectively. Differences were considered significant when *p* < 0.05.

## Results

### Design of the Microfluidic Chip

**FIGURE 1:**
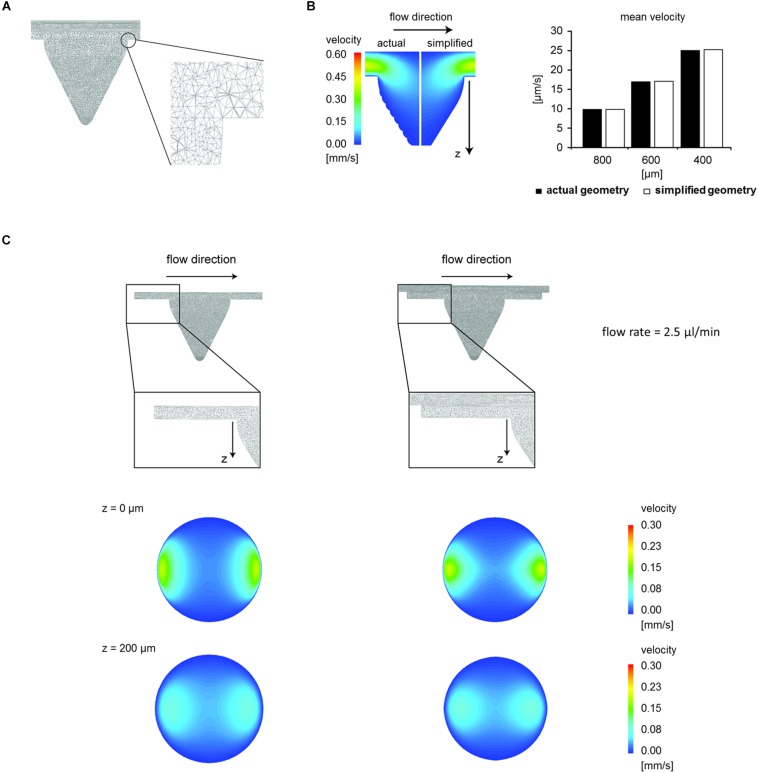
Optimization of the microfluidic chip design. **(A)** Geometric discretization of the concave chamber used in the computational model. Section of the mesh corresponding to the longitudinal mirror plane, and zoom of a mesh detail. **(B)** Colorimetric map of velocity magnitude relative to the longitudinal mirror plane and comparison between mean velocity values computed within the simplified and the actual chamber geometry at different depths (flow rate 2.5 μl/min). **(C)** Comparison between two different configurations of the chip chamber (i.e., without or with a 0.2 mm-high groove). Meshing scheme and colorimetric maps of velocity magnitude relative to cross-sections of the concave chamber at different depths (flow rate 2.5 μl/min).

In CFD simulations, tetrahedral meshing schemes with up to 2,000,000 elements were adopted (Figure 1A). To study the local fluid dynamics of the microfluidic device while preserving computational time, smaller elements were used to discretize the volume of the chamber, in which cells are seeded, while larger tetrahedrons were employed for the microchannels, designed for cell loading. CFD modeling was applied to validate the use of a simplified chamber geometry (i.e., without micro-burrs deriving from micro-machining) instead of the actual chamber geometry in the simulations. No relevant differences were found in the velocity field at any depths between the simplified and the actual geometry at a flow rate equal to 2.5 μl/min (Figure 1B, average velocity difference 0.6%). Hence, the simplified chamber geometry was used in all the subsequent CFD simulations.

**FIGURE 2:**
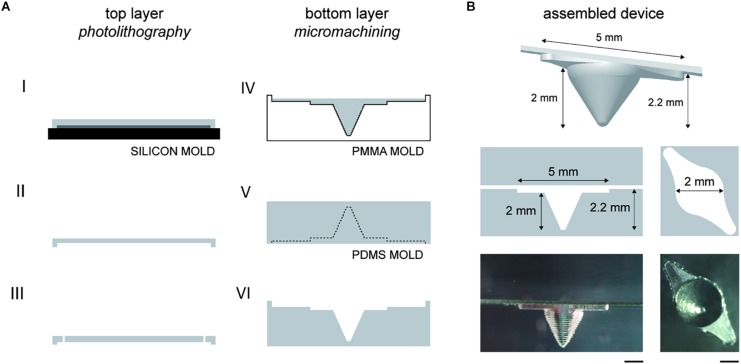
Microfluidic chip fabrication. **(A)** Schematic of the fabrication process. The top layer mold was obtained by transferring the channel layout onto a silicon wafer by photolithography. PDMS was casted and cured (I). The PDMS layer was detached (II) and inlet/outlet ports were punched (III). The bottom layer was obtained through multiple fabrication steps. The chamber and the groove were machined on a PMMA substrate with a CNC milling machine. PDMS was poured on the PMMA mold and cured (IV). Subsequently, PDMS was detached and used as mold for PDMS casting (V). The PDMS layer was then detached (VI) and bonded to the top layer. **(B)** 3D rendering and lateral view of the assembled microfluidic device and top view of the concave chamber (scale bars: 1 mm).

Subsequently, starting from the simplified chamber geometry, two computational models were generated with and without the groove upstream and downstream the chamber (Figure 1C). We demonstrated that the introduction of a 200 μm-deep groove reduced the flow velocity at different depths of the microchamber. Indeed, at a 2.5 μl/min flow rate, the average velocity at the top of the microchamber (*z* = 0 μm) changed from 46.34 μm/s without groove to 38.17 μm/s with groove (−18%). The average velocity decreased also at *z* = 200 μm from 35.37 μm/s without groove to 33.87 μm/s with groove (−4.2%). Based on the results of CFD optimization, the microfluidic chip was fabricated including the upstream and downstream groove (Figure 2A). The 3D rendering and geometrical features of the assembled device are illustrated in Figure 2B.

### Cell Trapping Experiments

**FIGURE 3:**
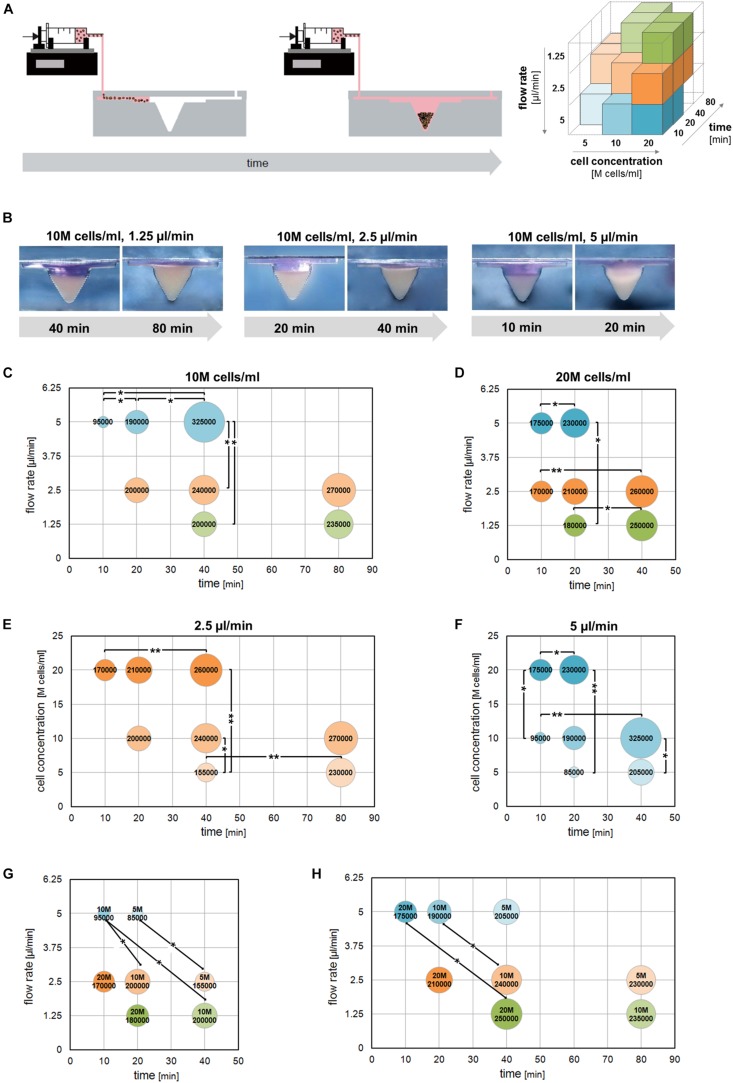
Cell trapping analysis. **(A)** Schematic showing the experimental set-up. The tested experimental conditions are represented as a 3D matrix. **(B)** Representative side views showing the progressive cell trapping in the chamber for different cell concentrations and flow rates. **(C–H)** Bubble graphs showing the average number of cells trapped in the chamber in correspondence of specific combinations of cell concentrations, flow rates, and seeding times. The bubble size is proportional to the number of trapped cells indicated. **(C,D)** Number of trapped cells obtained using different flow rates and seeding times starting from 10M or 20M cells/ml. **(E,F)** Number of trapped cells obtained for different cell concentrations and seeding times applying 2.5 or 5 μl/min. **(G,H)** Number of trapped cells obtained using parameter combinations theoretically leading to the same outcome. All the experimental conditions were tested independently at least six times (*p < 0.05; **p < 0.01).

Based on CFD optimization, cell trapping experiments were performed to demonstrate that tuning multiple non-geometrical parameters allows controlling the number of trapped cells. An overview of the tested combinations is displayed as 3D matrix (Figure 3A).

The gradual filling of the chamber during cell seeding is shown in Figure 3B. The number of trapped cells starting from cell suspensions containing 10M and 20M cells/ml gradually increased over time for each single flow rate. However, a non-linear increase of the trapped cell number was observed when comparing different flow rates at the same time or the same flow rate at different times (Figures 3C,D). For instance, seeding 10M cells/ml at 1.25 μl/min for 40 min resulted in 200,000 trapped cells, but the application of twofold and fourfold higher flow rates (i.e., 2.5 and 5 μl/min) only led to increases of + 20 and + 62.5% in the trapped cell number, respectively. Similarly, seeding 10M cells/ml at 2.5 μl/min for 20 min resulted in 200,000 trapped cells, but using two- and fourfold higher seeding times (i.e., 40 and 80 min) only led to respective increases of + 20 and + 35% in the number of trapped cells. Similar results were obtained by grouping cell trapping data for specific flow rates (Figures 3E,F). Indeed, within each single cell concentration, the trapped cell number increased over time for both 2.5 and 5 μl/min flow rates, but in most cases, this increase was not directly proportional to the increase in time. To better represent this phenomenon, we grouped together the data derived from experimental conditions theoretically expected to result in an equivalent trapped cell number (Figures 3G,H). Comparison between experimental groups with the same cell concentration but different flow rates and seeding times revealed that cell seeding at the highest flow rate (i.e., 5 μl/min) applied for a short time corresponded to a lower number trapped cells compared to lower flow rates used for a longer time. Conversely, no relevant differences were found between experimental groups where 1.25 and 2.5 μl/min flow rates were applied. The aforementioned results demonstrated that the interplay among different parameters, such as cell concentration, flow rate, and seeding time modulates the number of cells trapped into the seeding chamber.

### Generation of a Predictive Model for Cell Trapping

**FIGURE 4:**
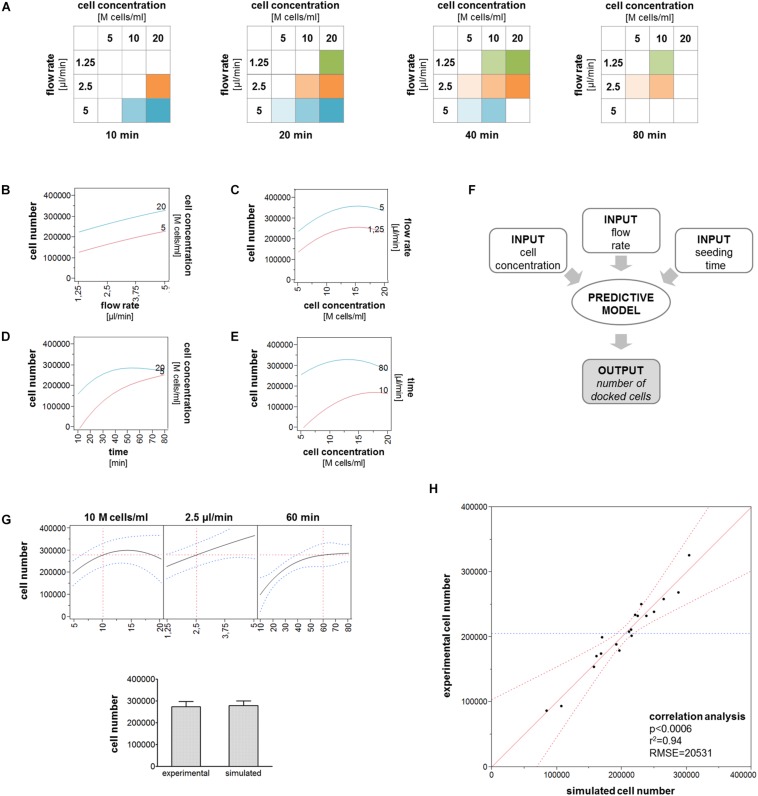
Cell trapping model. **(A)** All the experimental conditions included in the model are represented as 2D matrices. **(B–E)** Interaction plots describing the relation between cell concentration and flow rate or seeding time. Lines describe the variation of the number of trapped cells in correspondence of the minimum and maximum values of the variable indicated in the right *y*-axis for increasing values of the variable indicating in the *x*-axis. **(F)** Schematic of the working principle of the predictive model. **(G)** Comparison between the numbers of trapped cells obtained experimentally and predicted by the model for the following parameters: 10M cells/ml, 2.5 μl/min, 60 min. **(H)** Correlation plot describing the correlation between the number of trapped cells experimentally measured and the number predicted by the model.

Multiple interaction plots were drawn to describe the interactions between cell concentration and either the flow rate or the seeding time (Figure 4A). The interaction plots between cell concentration and flow rate generated parallel curves indicating that the trapped cell number increased similarly with increasing flow rates for 5M and 20M cells/ml (Figure 4B) and that the trapped cell number increased similarly with increasing cell concentrations at both 1.25 and 5 μl/min (Figure 4C). Differently, non-parallel curves were obtained by the correlation of seeding time and cell concentration. In particular, the trapped cell number gradually increased over time when using a low cell concentration (5M cells/ml), whereas it reached a plateau with a high cell concentration (20M cells/ml) (Figure 4D). Accordingly, approximately the same number of cells trapped in the chamber after a long seeding phase (80 min) independently from the starting cell concentration, while the cell concentration correlated with the number of trapped cells after a short seeding phase (10 min) (Figure 4E). The cell trapping data obtained by testing multiple combinations of these three parameters were used to generate a model able to predict the number of trapped cells (Figure 4F). We tested the model with specific combinations of input parameters, and we found that the values predicted by the model were similar to the experimental values, as shown in the example in Figure 4G. Furthermore, the correlation analysis demonstrated that simulated cell number values obtained by testing the model with all the experimental input combinations significantly correlated with the values measured in our experiments (Figure 4H).

### Generation of 3D Cell Spheroids

**FIGURE 5:**
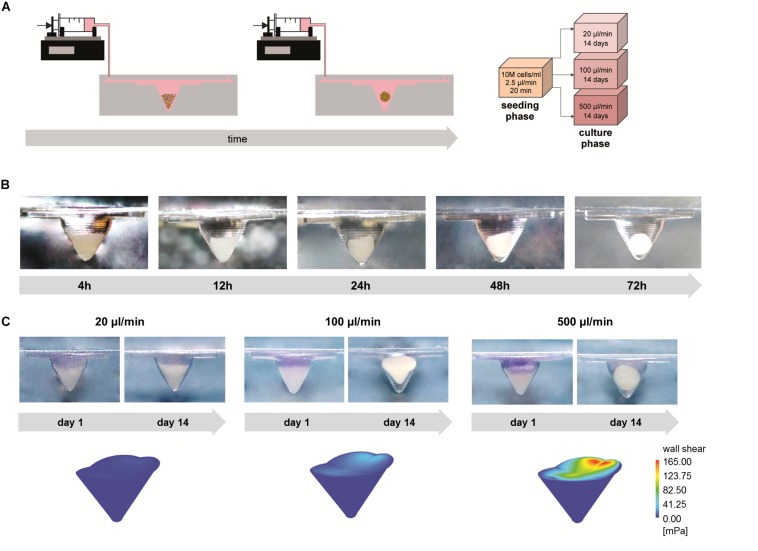
3D cell spheroid formation under different perfusion conditions. **(A)** Schematic showing the experimental set-up and the tested experimental conditions. **(B)** Side view of the concave chamber showing aggregate formation over time. **(C)** Representative pictures showing the process of spheroid formation and colorimetric maps of shear stresses applied on the concave chamber filled with cells under different perfusion conditions (scale bars: 1 mm).

Based on the cell trapping results, the microfluidic devices were seeded with 10M cells/ml at 2.5 μl/min for 20 min to achieve 200,000 trapped cells in the chamber. Three conditions of intermittent medium perfusion (20, 100, or 500 μl/min for 1 min every hour) were applied for 14 days (Figure 5A). A representative time course describing cell aggregate generation is depicted in Figure 5B. Initially, the influence of different flow rates on the formation of 3D cell aggregates was evaluated. Application of a low flow rate (20 μl/min) did not result in the generation of stable 3D aggregates. Due to the impossibility to retrieve stable cell aggregates from chips perfused with 20 μl/min, these samples were excluded from the subsequent analyses. Culture under perfusion at 100 and 500 μl/min resulted in the formation of stable 3D cell aggregates (Figure 5C). In particular, a flow rate of 100 μl/min induced the formation of conical aggregates, while spherical aggregates formed at 500 μl/min. These results correlated with the different shear stress profiles exerted by the flow in the different perfusion conditions. CFD simulations were performed to compute wall shear stress profiles on the surface of the microchamber filled with cells. The maximum wall shear stress gradually increased from 6.31 (20 μl/min) to 32.12 mPa (100 μl/min) and reached a peak equal to 164.92 mPa at 500 μl/min flow rate. Similarly, the mean wall shear stress increased from 2.37 (20 μl/min) to 13.74 mPa (100 μl/min), reaching the highest value (63.89 mPa) at 500 μl/min.

### Culture and Analysis of 3D Cell Spheroids

**FIGURE 6:**
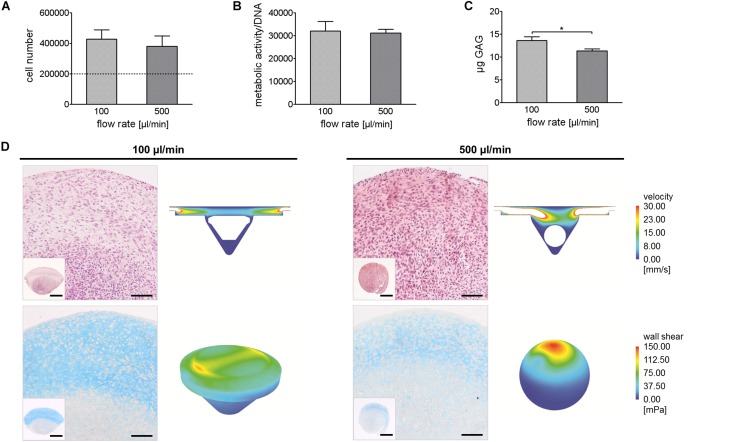
Comparison of 3D aggregates generated under different perfusion conditions. **(A–C)** Quantification of cell number, normalized metabolic activity, and GAG content in 3D articular chondrocyte spheroids after 14 days of culture (*p < 0.05). **(D)** Hematoxylin/eosin (upper images) and Alcian Blue (lower images) staining of spheroids cultured in perfusion for 14 days under different flow regimen and respective velocity fields and shear stress colorimetric maps (scale bars: image 100 μm; inset 500 μm).

We compared cell number, normalized metabolic activity, and GAG production in cell aggregates cultured at 100 and 500 μl/min. In both conditions, cell number increased after 14 days compared to day 0 (dotted line, Figure 6A). Cell number and normalized metabolic activity in aggregates perfused at 100 and 500 μl/min were comparable (Figures 6A,B), while GAG production resulted to be higher in cell aggregates perfused at 100 μl/min compared to 500 μl/min (Figure 6C). Accordingly, in aggregates cultured at 100 μl/min, an abundant GAG deposition was observed, even though restricted to the upper region of the disk-shaped aggregate, which was directly exposed to the flow. In this region, cellularization was inferior compared to the lower region of the aggregate. In aggregates perfused at 500 μl/min, cells were more evenly distributed through the aggregate. Only in the upper region of the pellet, which was directly exposed to the medium flow, it was possible to detect a positive, yet weak, staining for GAGs.

To explain these data, the two perfusion conditions were analyzed through CFD modeling. Two different models were applied to resemble the conical (Ø_*max*_ 1.9 mm, height 1.1 mm) and the spherical (Ø 1 mm) cell aggregate generated at 100 and 500 μl/min, respectively (Figure 6D). Computational simulations allowed predicting velocity and wall shear stress profiles when aggregates were exposed to 100 or 500 μl/min flow rate. Consistently, fluid velocity was lower in the chamber exposed to 100 μl/min flow rate compared to the chamber exposed to 500 μl/min flow rate, reaching max values equal to 53.53 and 260.25 mm/s, respectively. As expected, in both conditions, the highest wall shear stress values were predicted in correspondence of the surfaces directly exposed to the flow. Remarkably, despite similar peak values were measured for the disk-shaped and the spherical aggregates (127.57 and 148.83 mPa, respectively), the upper surface of the disk-shaped aggregate was characterized by a more homogeneous wall shear stress distribution compared to that of the spherical aggregate. Indeed, the disk-shaped pellet showed two peek values of shear stress on lateral regions of its upper side (max 127.57 mPa, mean 34.37 mPa), while the spherical pellet showed a singular peak value of shear stress on its upper side (max 148.83 mPa, mean 18.38 mPa).

## Discussion

The use of 3D spheroids allows overcoming the boundaries of standard 2D cell cultures by creating environments more similar to biological tissues (Pampaloni et al., 2007; Fang and Eglen, 2017). Microfluidics enables the creation of highly controlled environments, reducing the use of cells and reagents and the associated costs, and allowing high-throughput analysis (Whitesides, 2006). For these reasons, in recent years, efforts have been made to integrate the generation and the culture of 3D spheroids in a single device, with promising outcomes in different fields (Brady et al., 2015; Lim and Park, 2018; Mulholland et al., 2018; Ko et al., 2019).

In view of the on-chip generation and culture of 3D spheroids, one of the main objectives is to achieve consistent cell deposition (Kang et al., 2010). This means that the method used for cell trapping and for the subsequent spheroid formation has to be reproducible, robust, and fully predictable. Furthermore, to aim for the routine application in the laboratory practice, ease-of-use of the microfluidic device should be pursued. For what concerns the output predictability, CFD modeling provides an invaluable tool to couple the local fluid dynamics with experimental outcomes which can be exploited both to rationally design the microfluidic device and to understand the influence of mechanical stimulation on cellular behavior.

In this study, we designed and tested a microfluidic device that allows obtaining spheroids with controlled cell number and culturing them in dynamic conditions. Here, we applied an integrated computational–experimental approach in all the phases of the study, from the design of the device to the interpretation of biological results.

Starting from standard approaches to obtain 3D spheroids by cell centrifugation, the chamber design was built with a conical geometry resembling 1.5 ml microcentrifuge tubes. In the design phase, CFD simulations were used to optimize the geometry of the microfluidic device by adding a groove before and after the conical chamber. This modification allowed modifying flow velocity field to favor cell trapping in the conical chamber. Indeed, as reported in previous studies, the combination of device geometry and flow velocity is a crucial factor for the improvement of cell trapping efficiency (Manbachi et al., 2008; Cioffi et al., 2010). However, our results showed that, given a combination of cell concentration, seeding time, and flow rate expected to result in the same number of trapped cells, increasing the fluid velocity above a certain threshold negatively affects cell trapping. In particular, when fluid velocity is higher, cells are dragged by the flow to continue their path along the channel balancing the effect of gravity force and resulting in a lower number of cells trapped within the conical chamber. These results are partially in contrast with a previous study which demonstrated that increasing the seeding flow rate in a microfluidic device with U-shaped collecting traps, it was possible to push back cells that, with lower flow rates, tended to escape from the traps before adhering to each other (Wu et al., 2008). This discrepancy indicates that fluid-dynamic assumptions cannot be easily generalized from one device to another, since the device geometry is determinant. On the other hand, our results showed that even seeding cells with low fluid velocity, it is possible to fill the conical chamber with cells, as also reported in a previous microfluidic model where gravity-driven flow was used to let cells settle in the cell culture chambers (Patra et al., 2013).

Cell trapping experiments based on the combination of cell concentration, flow rate, and seeding time were used to build a model able to predict the synergic effect of multiple non-geometrical parameters on the number of trapped cells. Using specific combinations of input parameters, the model was validated demonstrating its ability to predict the number of trapped cells obtained experimentally, thus supporting its use as a tool to plan new experiments saving time and resources in the optimization phase.

The conical chamber configuration proved to be suitable to support cell spheroid formation within 24 h after cell seeding, due to the synergic action of fluid flow and gravity sedimentation. Additionally, our device allowed the biophysical stimulation of the developing constructs, which resulted in the formation of stable cell spheroids, when adequate levels of shear stress were applied. Indeed, very low shear stress levels did not yield the formation of a stable spheroid. This result indicates that in our system cell trapping *per se* was not sufficient to induce stable cell aggregation, not even in the case of ACs that are intrinsically prone to form 3D aggregates. On the other hand, the other flow rates tested (i.e., 100 and 500 μl/min) were able to promote spheroid formation. Noticeably, even when cultured with the highest flow rate, the maximum shear stress (148.83 mPa) imposed on cells was well below 1 Pa, the threshold above which shear stress loses the chondroprotective potential and begins to be detrimental for cell survival (Mohtai et al., 1996; Wang et al., 2010; Gharravi et al., 2014). Interestingly, as expected, shear stress distribution on trapped cells was similar for both flow rates, with maximum shear values found in the top surface of cell aggregate on the opposite side from the inlet channel. However, culturing trapped cells with different flow rates allowed creating cell spheroids characterized by different size and shape, indicating that these features are strictly dependent on fluid dynamic parameters in our system. Indeed, lower shear stress values led to bigger and less rounded spheroids, while higher shear stress values led to smaller and more rounded spheroids. We hypothesized that this phenomenon was due to cell response to the fluid dynamic stimulation in the attempt to minimize shear stresses on pellet surface (Ota et al., 2011). Our hypothesis was confirmed by the hematoxylin and eosin staining. When spheroids were cultured with a low flow rate (i.e., 100 μl/min), cells tended to migrate toward the core region of the pellet forming a cell gradient, while when spheroids were cultured with a high flow rate (i.e., 500 μl/min), cells tended to be more evenly distributed and form more compact pellets. Finally, our results demonstrated that fluid dynamic stimulation plays a very important role also after spheroid formation influencing GAG deposition. These results are not unexpected, since in several studies, biophysical cues have been applied to stimulate 3D constructs through micro- and macro-scale systems, demonstrating their effects on chondrocyte phenotype (Raimondi et al., 2011; Mayer et al., 2016; Salinas et al., 2018; Occhetta et al., 2019; Sharifi and Gharravi, 2019). Alcian blue staining showed that, regardless of the flow rate, the pellet region in direct contact with culture medium was characterized by a higher amount of GAGs. This result is in accordance with several studies showing that AC metabolism is directly modulated by shear forces in a wide range of intensity (i.e., 0.01–1 Pa) acting on the cells through mechanotransduction processes and inducing the upregulation of cartilage-specific ECM components, such as GAG and other proteoglycans, as recently reviewed (Sharifi and Gharravi, 2019). It must be noted that the ACs used in this study were derived from osteoarthritic donor, albeit only macroscopically non-fibrillated regions of cartilage were harvested for cell isolation. This means that the redifferentiation potential of our cells may be lower compared to ACs obtained from healthy donors. However, our experience with osteoarthritic chondrocytes indicates that, when cultured in the presence of suitable growth factors combinations, these cells are able to redifferentiate and produce cartilage-specific matrix, in line with literature reports that indicate this cell source as suitable for cartilage tissue-engineering applications (Stoop et al., 2007; Hsieh-Bonassera et al., 2009; Lopa et al., 2013). Based on the similar effects induced by fluid flow on ACs derived from the most diverse sources (Sharifi and Gharravi, 2019), we can reasonably speculate that although absolute amounts of GAG produced by healthy and osteoarthritic chondrocytes may differ, a similar outcome can be expected in cell response to biophysical stimulation. The observed results may either depend on a direct effect of flow rate on GAG production or be the result of a combination of biophysical and biochemical stimuli. In fact, the supply of nutrients to cells *in vitro* is controlled largely by diffusion. For this reason, mass transfer of nutrients within static culture systems is limited, while it is increased by fluid motion in dynamic culture systems (Freed et al., 1994; Vunjak-Novakovic and Freed, 1998). In accordance with a previous study investigating the effect of fluid flow on chondrogenic differentiation of human mesenchymal stem cells (MSC) (Kock et al., 2014), it is possible that less compact and more porous pellets obtained with the lowest flow rate favor nutrient and oxygen diffusion toward the inner core, thus explaining the higher amount of GAG found in the spheroids cultured with the lowest flow rate. On the contrary, more compact and less porous pellets obtained with the highest flow rate may limit nutrient and oxygen diffusion toward the inner core, despite the presence of a higher flow rate. Additionally, increasing the flow rate could cause a superior wash-out of external GAG molecules as observed in several microfluidic and non-microfluidic systems (Mizuno et al., 2001; Saini and Wick, 2003; Villanueva et al., 2009; Kock et al., 2014). This hypothesis could not be confirmed since the quantification of GAG release in the medium was not planned in our experimental set-up. Indeed, the amount of GAG released in the medium was expected to be under the detection limit of standard assays, considering the volume of medium perfused in the chamber during culture and the limited number of cells contained in the pellet. However, based on the supporting evidences provided by the literature, we hypothesize that this factor can at least partially explain the lower amount of GAG observed in correspondence of the highest flow rate applied.

## Conclusion

The described device meets the requirements for an easily accessible and consistent process to generate and culture 3D cell spheroids in a microfluidic set-up that could be upscaled to increase the throughput of the system. Our system allowed predicting the dimension and shape of the generated spheroid, proving to be highly reproducible and flexible. The particular advantage of the current system is the possibility to achieve different outcomes by simply tuning non-geometrical parameters without the need to modify the chip design. Considering the wide application of 3D cell spheroids, this platform holds a great potential both to generate *in vitro* models related to different research fields and to produce mature building blocks for tissue biofabrication.

## Data Availability Statement

The datasets generated for this study are available on request to the corresponding author.

## Ethics Statement

Ethical review and approval was not required for the study on human participants in accordance with the local legislation and institutional requirements. The patients/participants provided their written informed consent to participate in this study.

## Author Contributions

FP, MP, MR, and MM contributed to the conception and design of the study. SL, FP, GT, and MP performed the experiments and analyzed the data. SB and VM performed the computational analysis and analyzed the relative data. LZ provided the cartilage biopsies for articular chondrocyte isolation. SL, FP, GT, and VM wrote the manuscript. All authors contributed to manuscript revision and read and approved the submitted version.

## Conflict of Interest

The authors declare that the research was conducted in the absence of any commercial or financial relationships that could be construed as a potential conflict of interest.
